# Efficacy of budesonide/glycopyrronium/formoterol metered dose inhaler in patients with COPD: post-hoc analysis from the KRONOS study excluding patients with airway reversibility and high eosinophil counts

**DOI:** 10.1186/s12931-021-01773-1

**Published:** 2021-06-28

**Authors:** Shigeo Muro, Hisatoshi Sugiura, Patrick Darken, Paul Dorinsky

**Affiliations:** 1 Department of Respiratory Medicine, Nara Medical University Graduate School of Medicine, 840 Shijo-cho, Kashihara-shi, Nara, 634-8521 Japan; 2 Department of Respiratory Medicine, Tohoku University Graduate School of Medicine, Sendai, Japan; 3 AstraZeneca, Wilmington, DE USA; 4 AstraZeneca, Durham, NC USA

**Keywords:** COPD, Asthma-like features, Triple therapy, Budesonide, Glycopyrrolate, Formoterol fumarate, KRONOS, Pulmonary function, Exacerbation

## Abstract

**Background:**

In the Phase III KRONOS study, triple therapy with budesonide/glycopyrronium/formoterol fumarate metered dose inhaler (BGF MDI) was shown to reduce exacerbations and improve lung function versus glycopyrronium/formoterol fumarate dihydrate (GFF) MDI in patients with moderate-to-very severe chronic obstructive pulmonary disease (COPD). However, whether the benefits related to the ICS component of BGF are driven by patients with high blood eosinophil counts (EOS) and/or airway reversibility has not been previously studied.

**Methods:**

KRONOS was a Phase III, double-blind, parallel-group, multicenter, randomized, controlled study of patients with moderate-to-very-severe COPD. Patients were randomized 2:2:1:1 to receive BGF 320/14.4/10 μg, GFF 14.4/10 μg, budesonide/formoterol fumarate dihydrate (BFF) MDI 320/10 μg via a single Aerosphere inhaler, or open-label budesonide/formoterol fumarate dihydrate dry powder inhaler 400/12 μg (BUD/FORM DPI; Symbicort Turbuhaler) twice-daily for 24 weeks. Efficacy outcomes included in this post-hoc analysis were change from baseline in morning pre-dose trough FEV_1_ over weeks 12–24 and the rate of moderate-to-severe and severe COPD exacerbations. Adverse events in the non-reversible subgroup are also reported.

**Results:**

Of 1896 patients analyzed, 948 (50%) were non-reversible and had EOS < 300 cells/mm^3^. In this group, BGF significantly improved morning pre-dose trough FEV_1_ versus BFF and BUD/FORM (least squares mean treatment difference, 95% confidence interval [CI] 69 mL [39, 99], unadjusted *p* < 0.0001 and 51 mL [20, 81], unadjusted *p* = 0.0011, respectively) and was comparable to GFF. BGF also significantly reduced annual moderate-to-severe exacerbation rates versus GFF (rate ratio [95% CI] 0.53 [0.37, 0.76], unadjusted *p* = 0.0005), with numerical reductions observed versus BFF and BUD/FORM. These results were similar for the overall study population. Safety findings were generally similar between non-reversible patients with EOS < 300 cells/mm^3^ and the overall population.

**Conclusions:**

In patients with moderate-to-very-severe COPD without airway reversibility and EOS < 300 cells/mm^3^, BGF significantly improved morning pre-dose trough FEV_1_ versus BFF and BUD/FORM and significantly reduced the rate of moderate-to-severe exacerbations versus GFF. These findings demonstrate that BGF can provide benefits for a broad range of patients with COPD, and that the overall findings of the KRONOS primary analysis were not driven by patients with reversible airflow obstruction or high eosinophil counts.

*Trial registration* ClinicalTrials.gov, NCT02497001. Registered 14 July 2015, https://clinicaltrials.gov/ct2/show/NCT02497001

**Supplementary Information:**

The online version contains supplementary material available at 10.1186/s12931-021-01773-1.

## Background

The Global Initiative for Chronic Obstructive Lung Disease (GOLD) recommends treatment with a triple combination of an inhaled corticosteroid (ICS), a long-acting muscarinic antagonist (LAMA), and a long-acting β_2_-agonist (LABA) for patients with COPD who experience further exacerbations despite LAMA/LABA or ICS/LABA therapy, or persistent breathlessness on ICS/LABA therapy [1]. However, the Japanese Respiratory Society (JRS) only recommends ICS-containing treatments for the management of patients with COPD with additional features associated with asthma (also known as asthma–COPD overlap or ACO), which include airway reversibility (post-bronchodilator response in forced expiratory volume in 1 s [FEV_1_] ≥ 200 mL and ≥ 12% from baseline values) and a blood eosinophil count (EOS) ≥ 300 cells/mm^3^ [2, 3]. GOLD acknowledges that asthma and COPD can share common clinical features, such as some degree of airway reversibility and high EOS, and recommends that pharmacotherapy should primarily follow asthma guidelines upon a concurrent diagnosis of asthma and COPD; however, pharmacological and non-pharmacological treatment of COPD may also be needed [1].

The KRONOS study (NCT02497001) was a 24-week, Phase III, randomized, double-blind, parallel-group trial, investigating the efficacy and safety of the triple fixed-dose combination budesonide/glycopyrronium/formoterol fumarate dihydrate metered dose inhaler (BGF MDI) versus the corresponding dual therapies glycopyrronium/formoterol fumarate dihydrate (GFF) MDI and, budesonide/formoterol fumarate dihydrate (BFF) MDI, and open-label budesonide/formoterol fumarate dihydrate dry powder inhaler (BUD/FORM DPI) in patients from Canada, China, Japan, and the US [4]. Treatment with BGF was shown to provide benefits on lung function, symptoms, and exacerbations versus dual therapies, and was well tolerated in patients with moderate-to-very severe COPD.

The efficacy of triple therapy has previously been reported in COPD populations that included patients with some features of asthma [5, 6]. Improvements in exacerbation rates following ICS therapy in COPD have been observed to occur over a broad range of blood EOS levels but with the magnitude of effect increasing as blood EOS increases [7]. However, lung function responses to ICS are driven by EOS in COPD [8] and benefits of ICS on lung function are greater in patients with asthma than COPD [9]. In this regard, it has also been suggested that the efficacy of ICS-containing treatments in COPD trials may be driven by patients with a history of asthma [5]. While KRONOS did not enroll patients with a current diagnosis of asthma, those with a previous history of asthma were not excluded, and some patients had certain disease characteristics which, while present in many patients with COPD, can also be clinical features of asthma (airway reversibility and/or elevated EOS) [1]. Therefore, this post-hoc analysis of the KRONOS study aimed to evaluate lung function and exacerbations in patients with moderate-to-very severe COPD who did not have airway reversibility and who had EOS < 300 cells/mm^3^, to assess whether the benefits of BGF were driven by patients with some clinical features that overlap with asthma.

## Methods

### Study design

Details of the KRONOS study design have been previously reported [4]. Patients were randomized 2:2:1:1 to receive BGF 320/14.4/10 μg, GFF 14.4/10 μg, or BFF 320/10 μg via a single Aerosphere inhaler, or open-label BUD/FORM DPI 400/12 μg (Symbicort Turbuhaler) for 24 weeks of twice-daily treatment. As BFF MDI is not an approved therapy for COPD, BUD/FORM DPI was included in the study as an approved active comparator for BGF MDI. As the administration instructions for DPIs and MDIs are markedly different, BUD/FORM DPI was administered in an open-label fashion to avoid the need for a double-dummy design that may have impacted proper device use*.* Of note, in BGF, GFF, and BFF the doses of glycopyrronium and formoterol fumarate dihydrate are equivalent to glycopyrrolate 18 μg and formoterol fumarate 9.6 μg, respectively.

The study was done in accordance with Good Clinical Practice, including the Declaration of Helsinki. The protocol and informed consent form were approved by appropriate institutional review boards or independent ethics committees. All patients provided written informed consent before screening.

### Study population

Inclusion criteria have been previously reported [4]. In brief, eligible patients were 40–80 years of age, were current/former smokers (smoking history of ≥ 10 pack-years), had moderate-to-very severe airflow limitation (post-bronchodilator FEV_1_ ≥ 25% and < 80% of predicted normal values using appropriate reference norms [e.g. for Japanese patients, JRS reference equations were used] [10, 11]), and a COPD Assessment Test (CAT) score ≥ 10, despite receiving ≥ 2 inhaled maintenance therapies for ≥ 6 weeks before screening. In addition, there was no requirement for a history of COPD exacerbations in the year before study entry.

Exclusion criteria included a current diagnosis of asthma, any clinically significant respiratory disease other than COPD, or any other clinically significant uncontrolled non-respiratory disease that could influence study results.

### Efficacy and safety outcomes

The primary and secondary endpoints have been reported [4]. The results presented here focus on the effects of the ICS component (budesonide) of BGF on the primary endpoint of trough FEV_1_ over weeks 12–24, and rate of moderate-to-severe and severe COPD exacerbations. Adverse events in the non-reversible subgroup are also reported.

### Statistical analyses

Efficacy data were analyzed in the modified intention-to-treat (mITT) population, which included data from all patients obtained prior to discontinuation from treatment. For this post-hoc analysis, outcomes were analyzed in patients without airway reversibility (change in FEV_1_ < 12% or < 200 mL after administration of albuterol) and with EOS < 300 cells/mm^3^. Results for the efficacy endpoints in all enrolled patients, i.e. with any level of airway reversibility and with no restriction on eosinophil count are also presented for reference.

Change from baseline in morning pre-dose trough FEV_1_ over weeks 12–24 was analyzed using a linear repeated measures model including treatment, visit, treatment by visit interaction, and ICS use at screening as categorical variables, and baseline FEV_1_, baseline eosinophil count, and percent reversibility to albuterol as continuous covariates.

The rate of moderate and/or severe exacerbations was analyzed using negative binomial regression, with adjustment for baseline post-bronchodilator percent-predicted FEV_1_ and baseline eosinophil count as continuous covariates and baseline COPD exacerbation history (0, 1, ≥ 2), country, and ICS use at screening as categorical covariates. Time at risk of experiencing an exacerbation was used as an offset variable in the model. As this was a post-hoc analysis, no adjustment was made for multiplicity for the subgroup analyses.

Safety variables were summarized descriptively in the safety population, which included all randomized patients who received at least one dose of treatment.

## Results

Half (948/1896) of the overall mITT population did not show reversibility to albuterol and had EOS < 300 cells/mm^3^. A total of 1071/1896 (56.5%) were non-reversible to albuterol and 822/1896 (43.4%) were reversible to albuterol; 112/1896 (5.9%) were reversible to albuterol and had EOS ≥ 300 cells/mm^3^. Of note, 1361/1896 (71.8%) patients were receiving ICS treatment at screening.

For patients who were non-reversible with EOS < 300 cells/mm^3^, demographic and disease characteristics were generally similar across treatment groups, and similar to that of the overall mITT population. Exceptions were median EOS and mean airway reversibility, which, as expected based on subgroup definition criteria, were greater in the overall population (Table 1). Patients who were non-reversible and had EOS < 300 cells/mm^3^ had a mean age of 65.7 years, 67.0% were male, 73.2% reported having no exacerbations in the previous 12 months, and 70.7% were using ICS at screening. In addition, the mean (standard deviation) post-bronchodilator FEV_1_/forced vital capacity ratio (FVC) was 0.48 (0.11) in non-reversible patients with EOS < 300 cells/mm^3^ and 0.49 (0.11) in the patients who were excluded from this analysis (due to reversibility and/or EOS ≥ 300 cells/mm^3^); FEV_1_/FVC ratio was 0.48 (0.11) for the overall population*.*

**Table 1:** Demographic and baseline disease characteristics (mITT population)

	BGF 320/14.4/10 µg		GFF 14.4/10 µg		BFF 320/10 µg		BUD/FORM 400/12 µg	
	Non-reversible and EOS < 300^b^ (*n* = 319)	Overall population (*n* = 639)	Non-reversible and EOS < 300^b^ (*n* = 315)	Overall population (*n* = 625)	Non-reversible and EOS < 300^b^ (*n* = 161)	Overall population (*n* = 314)	Non-reversible and EOS < 300^b^ (*n* = 153)	Overall population (*n* = 318)
Mean age, years (SD)	65.6 (7.5)	64.9 (7.8)	65.6 (7.6)	65.1 (7.7)	65.3 (7.2)	65.2 (7.2)	66.5 (7.5)	65.9 (7.7)
Male, *n* (%)	216 (67.7)	460 (72.0)	207 (65.7)	430 (68.8)	108 (67.1)	224 (71.3)	104 (68.0)	236 (74.2)
Mean CAT Score (SD)	18.4 (6.7)	18.7 (6.4)	18.2 (6.4)	18.1 (6.1)	18.5 (6.7)	18.4 (6.6)	17.6 (7.0)	18.0 (6.4)
Mean body mass index, kg/m^2^ (SD)	25.9 (6.9)	26.1 (6.7)	25.9 (6.5)	26.3 (6.4)	25.4 (5.4)	26.1 (5.8)	25.8 (6.0)	26.2 (6.3)
Current smoker, *n* (%)	123 (38.6)	256 (40.1)	132 (41.9)	257 (41.1)	61 (37.9)	115 (36.6)	64 (41.8)	122 (38.4)
Median number of pack-years smoked^a^ (range)	45.0 (10–192.5)	45.0 (10.0–256.0)	45.0 (10.0–171.0)	45.0 (10.0–171.0)	47.0 (10.0–192.0)	45.0 (10.0–192.0)	45.8 (10.0–153.0)	45.0 (10.0–180.0)
Median EOS, cells/mm^3^ (range)	130.0 (10.0–295.0)	150.0 (10.0–2815.0)	140.0 (15.0–295.0)	155.0 (15.0–2490.0)	125.0 (20.0–295.0)	152.5 (15.0–920.0)	140.0 (35.0–295.0)	150.0 (35.0–1100.0)
Exacerbation history, *n* (%)								
0	239 (74.9)	469 (73.4)	229 (72.7)	473 (75.7)	115 (71.4)	235 (74.8)	111 (72.5)	234 (73.6)
1	61 (19.1)	125 (19.6)	63 (20.0)	108 (17.3)	37 (23.0)	61 (19.4)	29 (19.0)	59 (18.6)
≥ 2	19 (6.0)	45 (7.0)	23 (7.3)	44 (7.0)	9 (5.6)	18 (5.7)	13 (8.5)	25 (7.9)
Post-bronchodilator FEV_1_% predicted (SD)	48.5 (15.1)	50.2 (14.3)	47.6 (14.3)	50.2 (13.8)	47.5 (14.4)	50.0 (14.0)	48.5 (14.0)	50.7 (13.8)
Mean reversibility, % (SD)	10.1 (8.7)	18.8 (14.4)	9.3 (9.0)	18.1 (14.3)	10.3 (9.4)	19.0 (16.5)	10.6 (8.2)	19.9 (15.1)
Use of ICS at screening, *n* (%)	220 (69.0)	464 (72.6)	226 (71.7)	447 (71.5)	117 (72.7)	225 (71.7)	107 (69.9)	225 (70.8)

Overall population data from [4]

BFF, budesonide/formoterol fumarate dihydrate; BGF, budesonide/glycopyrronium/formoterol fumarate dihydrate; BUD/FORM DPI, budesonide/formoterol fumarate dihydrate dry powder inhaler; CAT, COPD Assessment Test; COPD, chronic obstructive pulmonary disease; EOS, blood eosinophil count; FEV_1_, forced expiratory volume in 1 s; GFF, glycopyrronium/formoterol fumarate dihydrate; ICS, inhaled corticosteroid; MDI, metered dose inhaler; mITT, modified intent-to-treat; SD, standard deviation

^a^Number of pack years smoked = (number of cigarettes per day / 20) x number of years smoked

^b^Patients with airways not reversible to albuterol and EOS < 300 cells/mm^3^

### Efficacy

#### Lung function

In the overall mITT population, there were significant improvements in morning pre-dose trough FEV_1_ over weeks 12–24 for BGF versus GFF, BFF, and BUD/FORM (Table 2).

**Table 2:** Efficacy endpoints (mITT population; efficacy estimand)

	BGF 320/14.4/10 µg	GFF 14.4/10 µg	BFF 320/10 µg	BUD/FORM 400/12 µg
Change from baseline in morning pre-dose trough FEV_1_ (mL) over weeks 12–24				
Overall population				
*n*	592	559	278	288
LSM (SE)	138 (7.0)	118 (7.1)	61 (9.9)	76 (9.8)
BGF versus comparators				
LSM difference (95% CI)	–	20 (1, 39)	77 (53, 100)	62 (38, 85)
*p*-value	–	0.0424	< 0.0001	< 0.0001
Patients not reversible to albuterol, EOS < 300 cells/mm^3^				
*n*	285	277	138	140
LSM (SE)	97 (9.0)	102 (9.2)	28 (12.8)	46 (12.8)
BGF versus comparators				
LSM difference (95% CI)	–	–5 (–29, 20)	69 (39, 99)	51 (20, 81)
*p*-value	–	0.7041	< 0.0001	0.0011
Rate of moderate-to-severe exacerbations				
Overall population				
* n*	639	625	314	318
Patients with exacerbations, *n* (%)	108 (16.9)	157 (25.1)	65 (20.7)	61 (19.2)
Adjusted rate per year	0.46	0.95	0.56	0.55
BGF versus comparators				
Rate ratio (95% CI)	–	0.48 (0.37, 0.64)	0.82 (0.58, 1.17)	0.83 (0.59, 1.18)
*p*-value	–	< 0.0001	0.2792	0.3120
Patients not reversible to albuterol, EOS < 300 cells/mm^3^				
* n*	319	315	161	153
Patients with exacerbations, *n* (%)	56 (17.6)	80 (25.4)	37 (23.0)	39 (25.5)
Adjusted rate per year	0.46	0.87	0.56	0.68
BGF versus comparators				
Rate ratio (95% CI)	–	0.53 (0.37, 0.76)	0.81 (0.51, 1.29)	0.67 (0.43, 1.04)
*p*-value	–	0.0005	0.3770	0.0756
Rate of severe exacerbations				
Overall population				
* n*	639	625	314	318
Patients with exacerbations, *n* (%)	17 (2.7)	33 (5.3)	9 (2.9)	11 (3.5)
Adjusted rate per year	0.05	0.13	0.05	0.07
BGF versus comparators				
Rate ratio (95% CI)	–	0.36 (0.18, 0.70)	0.85 (0.34, 2.13)	0.69 (0.29, 1.61)
*p*-value	–	0.0026	0.7363	0.3861
Patients not reversible to albuterol, EOS < 300 cells/mm^3^				
* n*	319	315	161	153
Patients with exacerbations, *n* (%)	11 (3.4)	20 (6.3)	7 (4.3)	3 (2.0)
Adjusted rate per year	0.07	0.18	0.10	0.03
BGF versus comparators				
Rate ratio (95% CI)	–	0.40 (0.17, 0.94)	0.74 (0.24, 2.30)	2.10 (0.50, 8.81)
*p*-value	–	0.0365	0.6057	0.3096

Overall population moderate/severe data from [4]

Treatments were compared adjusting for baseline post-bronchodilator percent predicted FEV_1_ and baseline eosinophil count as continuous covariates and baseline COPD exacerbations history (0, 1, ≥ 2), country, and ICS use at screening as categorical covariates using negative binomial regression. Time at risk of experiencing an exacerbation was used as an offset variable in the model

BFF, budesonide/formoterol fumarate dihydrate; BGF, budesonide/glycopyrronium/formoterol fumarate dihydrate; BUD/FORM DPI, budesonide/formoterol fumarate dihydrate dry powder inhaler; CI, confidence interval; EOS, blood eosinophil count; FEV_1_, forced expiratory volume in 1 s; GFF, glycopyrronium/formoterol fumarate dihydrate; LSM, least squares mean; MDI, metered dose inhaler; mITT, modified intent-to-treat; SE, standard error

**Fig. 1:**
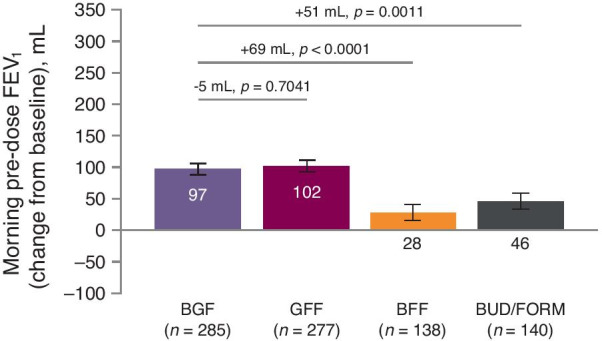
Change in morning pre-dose trough FEV_1_ over 12–24 weeks (patients with non-reversible airways and EOS < 300 cells/mm^3^). Bars are LSM (95% SE). Data analyzed in the mITT population using an efficacy estimand. Treatment comparisons are RR (95% CI). BFF, budesonide/formoterol fumarate dihydrate; BGF, budesonide/glycopyrronium/formoterol fumarate dihydrate; BUD/FORM, budesonide/formoterol fumarate dihydrate; FEV_1_, forced expiratory volume in 1 s; GFF, glycopyrronium/formoterol fumarate dihydrate; LSM, least squares mean; mITT, modified intent-to-treat; SE, standard error

In non-reversible patients with EOS < 300 cells/mm^3^, similar changes from baseline were observed in morning pre-dose trough FEV_1_ over weeks 12–24 with BGF and GFF (least squares mean [LSM], 95% confidence intervals [95% CI] 97 mL [80, 115] and 102 mL [84, 120], respectively; Fig. 1, Table 2). However, BGF significantly improved morning pre-dose trough FEV_1_ over weeks 12–24 compared with BFF and BUD/FORM (LSM treatment difference [95% CI] 69 mL [39, 99], unadjusted *p* < 0.0001 and 51 mL [20, 81], unadjusted *p* = 0.0011, respectively; Fig. 1, Table 2).

Data for the overall non-reversible population (all patients without reversibility to albuterol, regardless of eosinophil count) and the whole reversible population are presented in Additional file 1: Table S1. Across all treatments, greater improvements in lung function were observed in patients with reversibility compared to those without.

#### Moderate-to-severe exacerbations

**Fig. 2:**
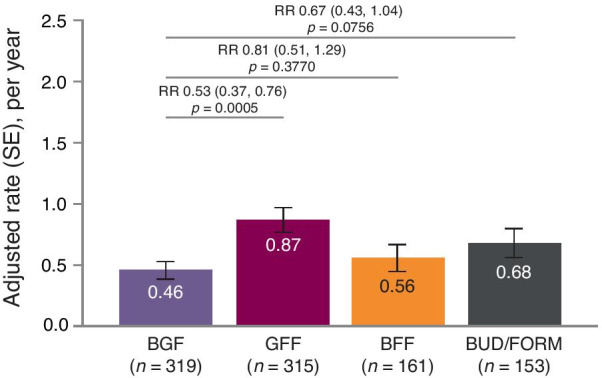
Adjusted rate of moderate or severe COPD exacerbations (patients with non-reversible airways and EOS < 300 cells/mm^3^). Bars are adjusted rate (± SE). Treatment comparisons are RR (95% CI). Data analyzed in the mITT population using an efficacy estimand. BFF, budesonide/formoterol fumarate dihydrate; BGF, budesonide/glycopyrronium/formoterol fumarate dihydrate; BUD/FORM, budesonide/formoterol fumarate dihydrate; CI, confidence interval; COPD, chronic obstructive pulmonary disease; EOS, blood eosinophil count; GFF, glycopyrronium/formoterol fumarate dihydrate; mITT, modified intent-to-treat; RR, rate ratio; SE, standard error

In the overall mITT population, BGF resulted in a significant reduction in annualized moderate-to-severe exacerbations versus GFF, and numerical reductions versus BFF and BUD/FORM (Table 2). In non-reversible patients with EOS < 300 cells/mm^3^, BGF significantly reduced annualized moderate-to-severe exacerbation rates versus GFF (adjusted rate ratio [RR], [95% CI] 0.53 [0.37, 0.76] *p* = 0.0005; Fig. 2; Table 2) and there were numerical reductions in annual moderate-to-severe exacerbation rates versus BFF and BUD/FORM (adjusted RR [95% CI] 0.81 [0.51, 1.29] and 0.67 [0.43, 1.04], respectively; Fig. 2; Table 2).

The pattern of changes in the annual rate of moderate-to-severe exacerbations was generally similar for the overall non-reversible and reversible subgroups, with significant benefit seen for BGF vs GFF in both populations. These findings are consistent with the changes observed overall and in non-reversible/EOS < 300 subgroups (Additional file 1: Table S1).

#### Severe exacerbations

In the overall population, BGF resulted in a nominally significant reduction in annualized severe exacerbations versus GFF, and numerical reductions versus BFF and BUD/FORM (Table 2). Similarly, BGF significantly reduced the rate of severe exacerbations versus GFF (adjusted RR [95% CI] 0.40 [0.17, 0.94], unadjusted *p* = 0.0365) in non-reversible patients with EOS < 300 cells/mm^3^, with numerical reductions versus BFF, but not versus BUD/FORM (RR [95% CI] 0.74 [0.24, 2.30] and 2.10 [0.50, 8.81], respectively; Table 2).

In the overall non-reversible population BGF numerically reduced the rate of severe exacerbations versus GFF but not BFF or BUD/FORM (Additional file 1: Table S1). In the reversible subgroup, BGF nominally significantly reduced the rate of severe exacerbations versus GFF and BUD/FORM and numerically reduced the rate of severe exacerbations versus BFF. However, it should be noted that there were very few severe exacerbation events in the non-reversible and reversible subgroups and the results should be interpreted with caution (Additional file 1: Table S1).

### Safety

Overall, safety findings in the overall population and the non-reversible with EOS < 300 cells/mm^3^ subgroup were similar (Table 3) and the most frequently reported treatment-emergent adverse events were nasopharyngitis, upper respiratory tract infection, COPD and bronchitis (Table 3). The incidence of confirmed pneumonia (number of patients [%]) was similar in the overall population (32 [1.7]) and the non-reversible with EOS < 300 cells/mm^3^ subgroup (17 [1.8]).

**Table 3:** Summary of TEAEs (safety population)

	BGF 320/14.4/10 µg		GFF 14.4/10 µg		BFF 320/10 µg		BUD/FORM 400/12 µg	
	Non-reversible and EOS < 300^b^ (*n* = 319)	Overall population (*n* = 639)	Non-reversible and EOS < 300^b^ (*n* = 315)	Overall population (*n* = 625)	Non-reversible and EOS < 300^b^ (*n* = 161)	Overall population (*n* = 314)	Non-reversible and EOS < 300^b^ (*n* = 153)	Overall population (*n* = 318)
TEAEs, *n* (%)								
≥ 1 TEAE	189 (59.2)	388 (60.7)	192 (61.0)	384 (61.4)	85 (52.8)	175 (55.7)	85 (55.6)	183 (57.5)
Treatment-related TEAEs^a^	51 (16.0)	112 (17.5)	51 (16.2)	91 (14.6)	20 (12.4)	48 (15.3)	17 (11.1)	40 (12.6)
TEAEs that led to early discontinuation	20 (6.3)	30 (4.7)	16 (5.1)	30 (4.8)	7 (4.3)	11 (3.5)	2 (1.3)	11 (3.5)
Serious TEAEs	27 (8.5)	55 (8.6)	36 (11.4)	68 (10.9)	10 (6.2)	21 (6.7)	9 (5.9)	29 (9.1)
Serious TEAEs related^a^ to study treatment	4 (1.3)	7 (1.1)	10 (3.2)	12 (1.9)	2 (1.2)	3 (1.0)	1 (0.7)	6 (1.9)
Serious TEAEs that led to early discontinuation	7 (2.2)	14 (2.2)	11 (3.5)	22 (3.5)	3 (1.9)	6 (1.9)	1 (0.7)	10 (3.1)
Deaths (all causes)	2 (0.6)	6 (0.9)	1 (0.3)	3 (0.5)	1 (0.6)	2 (0.6)	0	1 (0.3)
TEAEs occurring in ≥ 2% of patients with non-reversible airways and EOS < 300^b^ in any treatment arm, preferred term, *n* (%)								
Nasopharyngitis	28 (8.8)	49 (7.7)	17 (5.4)	41 (6.6)	11 (6.8)	26 (8.3)	16 (10.5)	30 (9.4)
Upper respiratory tract infection	28 (8.8)	65 (10.2)	24 (7.6)	38 (6.1)	8 (5.0)	18 (5.7)	7 (4.6)	22 (6.9)
COPD	11 (3.4)	17 (2.7)	20 (6.3)	32 (5.1)	6 (3.7)	8 (2.5)	4 (2.6)	13 (4.1)
Bronchitis	9 (2.8)	20 (3.1)	8 (2.5)	15 (2.4)	7 (4.3)	12 (3.8)	8 (5.2)	9 (2.8)
Dysphonia	8 (2.5)	20 (3.1)	3 (1.0)	5 (0.8)	6 (3.7)	15 (4.8)	4 (2.6)	6 (1.9)
Hypertension	8 (2.5)	13 (2.0)	5 (1.6)	10 (1.6)	4 (2.5)	8 (2.5)	3 (2.0)	4 (1.3)
Muscle spasms	11 (3.4)	21 (3.3)	1 (0.3)	8 (1.3)	4 (2.5)	17 (5.4)	4 (2.6)	6 (1.9)
Pneumonia	9 (2.8)	12 (1.9)	3 (1.0)	10 (1.6)	3 (1.9)	6 (1.9)	2 (1.3)	4 (1.3)
Back pain	1 (0.3)	8 (1.3)	9 (2.9)	12 (1.9)	2 (1.2)	4 (1.3)	4 (2.6)	8 (2.5)
Urinary tract infection	7 (2.2)	12 (1.9)	5 (1.6)	10 (1.6)	1 (0.6)	4 (1.3)	3 (2.0)	4 (1.3)
Dyspnea	7 (2.2)	9 (1.4)	4 (1.3)	9 (1.4)	1 (0.6)	8 (2.5)	1 (0.7)	8 (2.5)
Oral candidiasis	4 (1.3)	10 (1.6)	4 (1.3)	5 (0.8)	4 (2.5)	5 (1.6)	2 (1.3)	5 (1.6)
Pharyngitis	4 (1.3)	8 (1.3)	2 (0.6)	5 (0.8)	5 (3.1)	5 (1.6)	2 (1.3)	3 (0.9)
Herpes zoster	0	1 (0.2)	1 (0.3)	3 (0.5)	1 (0.6)	2 (0.6)	3 (2.0)	3 (0.9)

Overall population data from [4]

BFF, budesonide/formoterol fumarate dihydrate; BGF, budesonide/glycopyrronium/formoterol fumarate dihydrate; BUD/FORM DPI, budesonide/formoterol fumarate dihydrate dry powder inhaler; COPD, chronic obstructive pulmonary disease; EOS, blood eosinophil count; GFF, glycopyrronium/formoterol fumarate dihydrate; MDI, metered dose inhaler; TEAE, treatment-emergent adverse event

^a^Related = possibly, probably, definitely

^b^Patients with airways not reversible to albuterol and EOS < 300 cells/mm^3^

## Discussion

In this post-hoc analysis of the KRONOS study, the efficacy and safety of triple therapy with BGF 320/14.4/10 µg were evaluated in patients with moderate-to-very severe COPD who did not have reversibility to albuterol or EOS ≥ 300 cells/mm^3^, both of which are more common in patients with asthma than patients with COPD [1].

In comparison to guidelines for COPD, in which only certain patients are recommended to receive ICS-containing therapies, the Global Initiative for Asthma recommends ICS-containing therapies across all asthma severities [1, 12]. In some of the previous studies that reported benefits of triple therapy in patients with COPD, the inclusion of patients with a history of asthma has drawn criticism, with the specific concern that the observed treatment benefits may be driven by patients with asthma-like features [5, 6, 13]. However, in the KRONOS study, the benefits of ICS with BGF versus GFF were similar in patients with COPD either with or without potential asthma-like features. In both the overall population and patients whose airways were non-reversible to albuterol and who had EOS < 300 cells/mm^3^, moderate-to-severe exacerbation rates were significantly reduced for BGF compared with GFF by approximately 50% (for moderate-to-severe exacerbations) and 60% (for severe exacerbations). A similar numerical reduction was observed in patients whose airways were reversible to albuterol; moderate-to-severe exacerbation rates were reduced for BGF compared with GFF by 55% and by approximately 80% for severe exacerbations. Eosinophils are known to impact ICS efficacy outcomes in COPD. With respect to COPD exacerbations, reductions in exacerbation rates with an ICS are observed across a broad range of eosinophil levels, with greater reductions in exacerbation rates as eosinophil levels increase [1]. With respect to lung function, improvements with an ICS are also driven by eosinophils, and, as noted previously, the improvements are seen predominantly in patients with eosinophil levels > 250 cells/mm^3^ [4]. This likely explains why lung function improvements were similar for patients receiving BGF or GFF who were non-reversible to albuterol and had EOS < 300 cells/mm^3^.

BGF resulted in significant reductions in exacerbation rates versus GFF in patients with COPD without a history of asthma and without clinical features of asthma as assessed by non-reversibility to albuterol and EOS < 300 cells/mm^3^. This is notable as current JRS guidelines recommend ICS treatment be reserved for patients with evidence of both asthma and COPD [10]. Furthermore, while the GOLD report only recommends step-up from LAMA/LABA to triple therapy in patients with continuing exacerbations [1], significant benefits of BGF vs GFF were seen despite the fact that most patients in KRONOS did not have a history of exacerbations in the previous year (74%) [4, 14]. Therefore, the results of our study indicate that triple therapy may be beneficial for a wider population of patients with COPD than is reflected by current ACO or COPD guidelines and that the benefit of ICS on COPD exacerbations is not driven by patients with asthma-like features. The precise mechanisms underlying the reduction in exacerbations with BGF in patients without reversibility or high eosinophil levels are not known but may include enhanced bronchodilatory effects and/or anti-inflammatory effects with the triple combination relative to LAMA/LABA therapy [1, 15–18]. Further, in the KRONOS study, many patients had eosinophil levels above the threshold of approximately 75–100 cells/mm^3^ at which the beneficial effects of ICS on exacerbations are manifested [4, 19].

Several limitations of this work should be acknowledged. These include the sample size of the subgroup of patients analyzed who were non-reversible with EOS < 300 cells/mm^3^ (*n* = 948) and the 24-week duration of the study, which may be suboptimal when assessing COPD exacerbations and adverse events, such as pneumonia. Additionally, given their post-hoc nature, these subgroup analyses were not controlled for multiplicity. Nevertheless, we observed a clinically meaningful and significant difference between triple therapy and LAMA/LABA therapy in the subgroup of patients who were non-reversible with EOS < 300 cells/mm^3^ for both exacerbation endpoints.

## Conclusions

The findings of this post-hoc analysis of patients in the KRONOS study suggest that BGF can provide benefits for a broad range of patients with COPD, including those with no evidence of concurrent common traits or clinical features of asthma. Importantly, these findings also indicate that the benefit of ICS on reducing COPD exacerbations in KRONOS was not driven by subjects with some asthma-like features.

## Supplementary Information

**Additional file 1.** Efficacy endpoints among patients with or without reversibility to albuterol, regardless of eosinophil count (mITT population, efficacy estimand).

## Data Availability

Data underlying the findings described in this manuscript may be obtained in accordance with AstraZeneca’s data sharing policy described at https://astrazenecagrouptrials.pharmacm.com/ST/Submission/Disclosure.

## Abbreviations

ACO: Asthma–COPD overlap
BFF: Budesonide/formoterol fumarate
BGF: Budesonide/glycopyrronium/formoterol fumarate
BUD/FORM DPI: Budesonide/formoterol fumarate dihydrate dry powder inhaler
CAT: COPD Assessment Test
CI: Confidence interval
COPD: Chronic obstructive pulmonary disease
EOS: Blood eosinophil count
FEV_1_: Forced expiratory volume in 1s
GOLD: Global Initiative for COPD
GFF: Glycopyrronium/formoterol fumarate
ICS: Inhaled corticosteroids
JRS: Japanese Respiratory Society
LABA: Long-acting β_2_-agonists
LAMA: Long-acting muscarinic antagonists
LSM: Least squares mean
MDI: Metered dose inhaler
mITT: Modified intent-to-treat
RR: Rate ratio
SD: Standard deviation
SE: Standard error
TEAE: Treatment-emergent adverse event
