# Corrections to: Efficacy of budesonide/glycopyrronium/formoterol metered dose inhaler in patients with COPD: post-hoc analysis from the KRONOS study excluding patients with airway reversibility and high eosinophil counts

**DOI:** 10.1186/s12931-021-01803-y

**Published:** 2021-08-09

**Authors:** Shigeo Muro, Hisatoshi Sugiura, Patrick Darken, Paul Dorinsky

**Affiliations:** 1 Department of Respiratory Medicine, Nara Medical University Graduate School of Medicine, 840 Shijo-cho, Kashihara-shi, 634-8521 Nara, Japan; 2 Department of Respiratory Medicine, Tohoku University Graduate School of Medicine, Sendai, Japan; 3 AstraZeneca, Wilmington, DE USA; 4 AstraZeneca, Durham, NC USA

## Correction to: Respir Res (2021) 22:187 https://doi.org/10.1186/s12931-021-01773-1

The original version of this article unfortunately contained a mistake [1]. The minor correction of Table 1 was missing. That is, the text ‘FF’ should be ‘GFF’.

The correct Table 1 is given in this erratum.

**Table 1:** Demographic and baseline disease characteristics (mITT population)

	BGF 320/14.4/10 µg		GFF 14.4/10 µg		BFF 320/10 µg		BUD/FORM 400/12 µg	
	Non-reversible and EOS < 300^b^ (*n* = 319)	Overall population (*n* = 639)	Non-reversible and EOS < 300^b^ (*n* = 315)	Overall population (*n* = 625)	Non-reversible and EOS < 300^b^ (*n* = 161)	Overall population (*n* = 314)	Non-reversible and EOS < 300^b^ (*n* = 153)	Overall population (*n* = 318)
Mean age, years (SD)	65.6 (7.5)	64.9 (7.8)	65.6 (7.6)	65.1 (7.7)	65.3 (7.2)	65.2 (7.2)	66.5 (7.5)	65.9 (7.7)
Male, *n* (%)	216 (67.7)	460 (72.0)	207 (65.7)	430 (68.8)	108 (67.1)	224 (71.3)	104 (68.0)	236 (74.2)
Mean CAT Score (SD)	18.4 (6.7)	18.7 (6.4)	18.2 (6.4)	18.1 (6.1)	18.5 (6.7)	18.4 (6.6)	17.6 (7.0)	18.0 (6.4)
Mean body mass index, kg/m^2^ (SD)	25.9 (6.9)	26.1 (6.7)	25.9 (6.5)	26.3 (6.4)	25.4 (5.4)	26.1 (5.8)	25.8 (6.0)	26.2 (6.3)
Current smoker, *n* (%)	123 (38.6)	256 (40.1)	132 (41.9)	257 (41.1)	61 (37.9)	115 (36.6)	64 (41.8)	122 (38.4)
Median number of pack-years smoked^a^ (range)	45.0 (10–192.5)	45.0 (10.0–256.0)	45.0 (10.0–171.0)	45.0 (10.0–171.0)	47.0 (10.0–192.0)	45.0 (10.0–192.0)	45.8 (10.0–153.0)	45.0 (10.0–180.0)
Median EOS, cells/mm^3^ (range)	130.0 (10.0–295.0)	150.0 (10.0–2815.0)	140.0 (15.0–295.0)	155.0 (15.0–2490.0)	125.0 (20.0–295.0)	152.5 (15.0–920.0)	140.0 (35.0–295.0)	150.0 (35.0–1100.0)
Exacerbation history, *n* (%)								
0	239 (74.9)	469 (73.4)	229 (72.7)	473 (75.7)	115 (71.4)	235 (74.8)	111 (72.5)	234 (73.6)
1	61 (19.1)	125 (19.6)	63 (20.0)	108 (17.3)	37 (23.0)	61 (19.4)	29 (19.0)	59 (18.6)
≥ 2	19 (6.0)	45 (7.0)	23 (7.3)	44 (7.0)	9 (5.6)	18 (5.7)	13 (8.5)	25 (7.9)
Post-bronchodilator FEV_1_% predicted (SD)	48.5 (15.1)	50.2 (14.3)	47.6 (14.3)	50.2 (13.8)	47.5 (14.4)	50.0 (14.0)	48.5 (14.0)	50.7 (13.8)
Mean reversibility, % (SD)	10.1 (8.7)	18.8 (14.4)	9.3 (9.0)	18.1 (14.3)	10.3 (9.4)	19.0 (16.5)	10.6 (8.2)	19.9 (15.1)
Use of ICS at screening, *n* (%)	220 (69.0)	464 (72.6)	226 (71.7)	447 (71.5)	117 (72.7)	225 (71.7)	107 (69.9)	225 (70.8)

Overall population data from [4]

BFF, budesonide/formoterol fumarate dihydrate; BGF, budesonide/glycopyrronium/formoterol fumarate dihydrate; BUD/FORM DPI, budesonide/formoterol fumarate dihydrate dry powder inhaler; CAT, COPD Assessment Test; COPD, chronic obstructive pulmonary disease; EOS, blood eosinophil count; FEV_1_, forced expiratory volume in 1 s; GFF, glycopyrronium/formoterol fumarate dihydrate; ICS, inhaled corticosteroid; MDI, metered dose inhaler; mITT, modified intent-to-treat; SD, standard deviation

^a^Number of pack years smoked = (number of cigarettes per day/20) × number of years smoked

^b^Patients with airways not reversible to albuterol and EOS < 300 cells/mm^3^
