# Supplementary material for: Efficacy of budesonide/glycopyrronium/formoterol metered dose inhaler in patients with COPD: post-hoc analysis from the KRONOS study excluding patients with airway reversibility and high eosinophil counts
Source: Respir Res. 2021 Jun 28;22:187. doi: 10.1186/s12931-021-01773-1 (PMC8240276; doi:10.1186/s12931-021-01773-1)
Supplement: Supplementary file 1 — Additional file 1. Efficacy endpoints among patients with or without reversibility to albuterol, regardless of eosinophil count (mITT population, efficacy estimand). [file 12931_2021_1773_MOESM1_ESM.docx]

**Efficacy of budesonide/glycopyrronium/formoterol metered dose inhaler in patients with COPD: post-hoc analysis from the KRONOS study excluding patients with airway reversibility and high eosinophil counts**

Shigeo Muro^1^, Hisatoshi Sugiura^2^, Patrick Darken^3^, Paul Dorinsky^4^

^1^Department of Respiratory Medicine, Nara Medical University Graduate School of Medicine, Nara, Japan. ^2^Department of Respiratory Medicine, Tohoku University Graduate School of Medicine, Sendai, Japan. ^3^AstraZeneca, Wilmington, DE, USA, and ^4^AstraZeneca, Durham, NC, USA.

**Supplementary information**

**Table S1** Efficacy endpoints among patients with or without reversibility to albuterol, regardless of eosinophil count (mITT population, efficacy estimand)

|  | **BGF 320/14.4/10 µg** | **GFF 14.4/10 µg** | **BFF 320/10 µg** | **BUD/FORM 400/12 µg** |
| --- | --- | --- | --- | --- |
| **Change from baseline in morning pre-dose trough FEV_1_ (mL) over weeks 12–24** | | | | |
| *Patients not reversible to albuterol* |  |  |  |  |
| *n* | 315 | 314 | 159 | 161 |
| LSM (SE) | 97 (8.8) | 94 (8.8) | 25 (12.2) | 51 (12.2) |
| *BGF versus comparators* | | | | |
| LSM (95% CI) | - | 3 (–21, 27) | 72 (43, 101) | 46 (17, 75) |
| *p*-value | - | 0.8153 | <0.0001 | 0.0021 |
| *Patients reversible to albuterol* | | | | |
| *n* | 277 | 245 | 119 | 127 |
| LSM (SE) | 189 (11.2) | 147 (11.6) | 109 (16.3) | 111 (15.9) |
| *BGF versus comparators* | | | | |
| LSM (95% CI) |  | 42 (12, 73) | 81 (43, 119) | 78 (41, 115) |
| *p*-value |  | 0.0065 | <0.0001 | <0.0001 |
| **Rate of moderate-to-severe exacerbations** | | | | |
| *Patients not reversible to albuterol* | | | | |
| *n* | 353 | 357 | 184 | 177 |
| Patients with exacerbations, *n* (%) | 61 (17.3) | 89 (24.9) | 39 (21.2) | 44 (24.9) |
| Adjusted rate per year | 0.47 | 0.91 | 0.53 | 0.70 |
| *BGF versus comparators* | | | | |
| Rate ratio (95% CI) | - | 0.52 (0.36, 0.74) | 0.89 (0.56, 1.40) | 0.67 (0.44, 1.04) |
| *p*-value | - | 0.0003 | 0.6020 | 0.0749 |
| *Patients reversible to albuterol* | | | | |
| *n* | 286 | 266 | 130 | 140 |
| Patients with exacerbations, *n* (%) | 47 (16.4) | 67 (25.2) | 26 (20.0) | 17 (12.1) |
| Adjusted rate per year | 0.44 | 0.96 | 0.58 | 0.34 |
| *BGF versus comparators* | | | | |
| Rate ratio (95% CI) |  | 0.45 (0.30, 0.70) | 0.75 (0.43, 1.31) | 1.26 (0.69, 2.32) |
| *p*-value |  | 0.0003 | 0.3098 | 0.4510 |
| **Rate of severe exacerbations** | | | | |
| *Patients not reversible to albuterol* | | | | |
| *n* | 353 | 357 | 184 | 177 |
| Patients with exacerbations, *n* (%) | 14 (4.0) | 21 (5.9) | 7 (3.8) | 4 (2.3) |
| Adjusted rate per year | 0.08 | 0.16 | 0.08 | 0.04 |
| *BGF versus comparators* | | | | |
| Rate ratio (95% CI) | - | 0.51 (0.23, 1.13) | 1.00 (0.34, 2.90) | 2.09 (0.59, 7.40) |
| *p*-value | - | 0.0983 | 0.9953 | 0.2548 |
| *Patients reversible to albuterol* | | | | |
| *n* | 286 | 266 | 130 | 140 |
| Patients with exacerbations, *n* (%) | 3 (1.0) | 12 (4.5) | 2 (1.5) | 7 (5.0) |
| Adjusted rate per year | 0.00 | 0.01 | 0.00 | 0.02 |
| *BGF versus comparators* | | | | |
| Rate ratio (95% CI) |  | 0.17 (0.04, 0.66) | 0.63 (0.10, 4.13) | 0.16 (0.04, 0.66) |
| *p*-value |  | 0.0104 | 0.6307 | 0.0115 |

BFF, budesonide/formoterol fumarate dihydrate; BGF, budesonide/glycopyrronium/formoterol fumarate dihydrate; BUD/FORM DPI, budesonide/formoterol fumarate dihydrate dry powder inhaler; CI, confidence interval; FEV_1_, forced expiratory volume in 1 second; GFF, glycopyrronium/formoterol fumarate dihydrate; LSM, least squares mean; MDI, metered dose inhaler; mITT, modified intent-to-treat; SE standard error

Treatments were compared adjusting for baseline post-bronchodilator percent predicted FEV_1_ and baseline eosinophil count as continuous covariates and baseline COPD exacerbations history (0, 1, ≥2), country, and ICS use at screening as categorical covariates using negative binomial regression. Time at risk of experiencing an exacerbation was used as an offset variable in the model.
